# Method of 3D Coating Accumulation Modeling Based on Inclined Spraying

**DOI:** 10.3390/s24041212

**Published:** 2024-02-14

**Authors:** Danyang Yu, Chengzhi Su, Enguo Wang, Haifeng Bao, Fuheng Qu

**Affiliations:** 1 College of Mechanical and Electrical Engineering, Changchun University of Science and Technology, Changchun 130022, China; yudanyang@mails.cust.edu.cn (D.Y.); wangenguo@cust.edu.cn (E.W.); baohaifeng@cust.edu.cn (H.B.); 2 College of Artificial Intelligence, Changchun University of Science and Technology, Changchun 130022, China; 3 College of Computer Science and Technology, Changchun University of Science and Technology, Changchun 130022, China; qufh@cust.edu.cn

**Keywords:** repair by spraying, inclined spraying, 3D coating accumulation model

## Abstract

In the process of repairing the surface of products in aviation, aerospace, and other fields by spraying, accurate 3D cumulative-coating modeling is an important research issue in spraying-process simulation. The approach to this issue is a 3D cumulative-coating model based on inclined spraying. Firstly, an oblique spraying layer cumulative model was established, which could quickly collect the coating thickness distribution data of different spray distances. Secondly, 3D cumulative-coating modeling was conducted with the distance between the measuring point and the axis of the spray gun and the spraying distance between the measuring points as the input parameters, and the coating thickness of the measuring point as the output parameter. The experimental results show that the mean relative error of the cumulative model of the oblique spraying layer is less than 4.1% in the case of a 170~290 mm spraying distance and that the model is applicable in the range of −80~80 mm, indicating that the data on the oblique spraying coating proposed in this paper is accurate and fast. The accuracy of the 3D cumulative-coating model proposed in this paper is 1.2% and 21.5% higher than that of the two similar models, respectively. Therefore, the approach of 3D cumulative-coating modeling based on inclined distance spraying is discovered, demonstrating the advantages of fast and accurate modeling and enabling accurate 3D cumulative-coating modeling for spraying process simulation.

## 1. Introduction

At present, in the process of surface repair spraying of products in aviation, aerospace, and other fields, due to the fact that surface repair spraying is generally not repeated and the structure is complex, the qualified rate of spraying quality cannot rely on repeated tests to optimize the spraying process and can only rely on the prior knowledge of skilled technicians. Spraying process simulation is an important technical way to solve the optimization of such a spraying process, but the quality of the process simulation mainly depends on the accuracy of the process simulation model. Therefore, it is of great value to study the establishment of an accurate coating accumulation model.

Domestic and foreign scholars have conducted a lot of research on the coating accumulation model, which mainly focuses on three aspects: first, the infinite range of the Gaussian distribution model [1] and the Cauchy distribution model [2]. These models are only applicable to the spray gun vertical workpiece surface, so the application is less [3]. The second type is the limited range model, such as the piecewise function model [4], β distribution model [5], elliptical double β distribution model [6], uniform thickness model [7], etc. This type of model thinks that a spray gun is a conical spray and is widely used. The third type is to build a coating accumulation model by fitting experimental data, which is more in line with the actual spraying situation. The representative literature is shown in Table 1.

In [8], the experimental data were obtained by over-distance spraying, and the data were characterized by the β distribution function. Based on the BP neural network algorithm, a coating thickness distribution prediction model was established with the parameters of fitting the β distribution function as input parameters and the spray width, spray distance, and spray speed as output. The data acquisition method of the model has the problem of insufficient data density. The model only uses 15 sets of data for training, which affects the accuracy of the model. On the other hand, the β distribution function is not intuitive enough to express the data, and the information acquisition ability of the data is weak, so the parameters represented by it will also lose the accuracy of the model. In [9], a coating distribution model based on β distribution is proposed. By fitting the experimental data, the parameters, such as spraying flow rate and spraying distance, are introduced into the model, which improves the generalization ability of the model. In [10], based on the plane β distribution function, an inclined angle spraying model is established, and the model is used in the trajectory planning strategy to improve the uniformity of spraying. In [11], the β distribution model was used to determine the overlap width of the trajectory, and the coverage and uniformity of the coating were studied. The above literature uses the data acquisition method of fixed-distance spraying and the research on the coating deposition model based on the β-distribution model. The following lists their common shortcomings.

- The data are obtained by fixed-distance spraying. This method requires a large number of experiments, low data-acquisition efficiency, and insufficient data information.
- The β distribution model is relatively simple, and the ability to capture data features is weak. The coating thickness distribution model established by this method has low accuracy and weak adaptability.

**Table 1:** Reference information analysis table.

Reference	Modeling Method	Modeling Efficiency	Model Information Quantity	Model Accuracy	
				Maximum Error	Average Relative Error
Xue-mei, L. et al. [7]	β distribution model	The data density is insufficient, and the process of obtaining experimental data by fixed-distance spraying is complex.	The model is not intuitive enough to express the data, and the representation ability is weak.	43.7 μm	--
Guolei, W. et al. [8]	β distribution model		The spatial relationship of the model is considered, but the β distribution model is simple and the ability to capture data features is weak.	--	4.3%
Yi, W. et al. [11]	Elliptical double β distribution model	The data density is limited, and the collection efficiency is low for the data collection of flat plate fixed-distance spraying.	Compared with the β model, the elliptical double β model increases the complexity and expression ability of modeling, but the model established by fixed-distance spraying lacks the ability to adapt to any surface spraying.	2.8 μm	--
Shulin, Q. et al. [12]	Elliptical double β distribution model			3.7 μm	--
Yong, Z. et al. [13]	Parabolic distribution model	The fixed-distance experiment obtains data, and the modeling efficiency is low.	Considering the posture of the spray gun, and taking the spraying distance and spraying angle into account in the algorithm, it has a certain adaptability.	3.6 μm	--
Chen, C. et al. [14]	Gaussian distribution model	The data acquisition method of fixed-distance spraying is complex, and the data coverage is insufficient.	The model lacks spatial information expression.	--	25.2%
Wu, H. et al. [15]	Gaussian distribution model of 3D		Compared with the 2D Gaussian model, the 3D model can describe the coating accumulation more accurately and improve the accuracy of the model.	--	7.6%
Hua, R.-X. et al. [16]	Rectangular distribution model of 3D	The process of data acquisition is complex, and the amount of information is insufficient.	The 3D rectangular coating thickness distribution model is adopted, and the accuracy is high.	10.0 μm	--

In [12], based on the elliptic double β distribution function, a spray gun model with a description of the coating thickness distribution at the starting and ending positions of spraying was established. The model solves the problem of controlling the coating thickness at the junction of the trajectory. In [13], also based on the elliptical double β distribution model of static coating thickness, the distribution law of cumulative-coating thickness of spraying is analyzed. The study of the thickness of the interface area and the uniformity of the coating thickness are also problems that need to be solved in repair spraying. In [14], based on the elliptical double β distribution model of the static vertical spraying coating growth model of the spray gun, a variable flow coating cumulative thickness model was established. The model can determine the appropriate spraying flow rate according to any speed in the acceleration and deceleration section, thereby improving the coating thickness uniformity. The uniformity is increased by 10%. Compared with the β model, the elliptical double β model increases the complexity and expression ability of modeling, but the object of repair spraying is not only the plane. The experimental model established by fixed-distance spraying lacks the ability to adapt to any surface spraying. In [15], based on the elliptical double β model, a three-dimensional elliptical double β model is established by numerical optimization. The model is more complex than the two-dimensional model for characterizing the thickness value at a certain time. For complex surface spraying, the film thickness uniformity is increased by 26.9%. In [16], based on the parabolic model, the static spraying model of the variable attitude spray gun and the dynamic spraying model of the variable attitude spray gun along the arc path are established, respectively. The author adapts to the spraying of irregular planes by changing the attitude of the spray gun, and also takes the spraying height and spraying angle into account in the optimization of the algorithm, which has a certain adaptability. However, the method of data acquisition is obtained by a fixed-distance experiment, which affects the efficiency of modeling. In [17], the coating thickness model based on Gaussian distribution is established by using the data collected by fixed-distance dip angle spraying. The model does not consider the influence of spraying distance and lacks the expression of spatial information of the model. At the same time, the model also uses the data acquisition method of fixed-distance spraying. The experiment is complex, and the data coverage is insufficient. In [18], the 3D coating accumulation model is also established based on the Gaussian distribution model. Compared with the 2D Gaussian model, the 3D model can describe the coating accumulation more accurately and improve the accuracy of the model. However, the data acquisition method of the model has the same problem as [17]. In [19], according to the application requirements, the rectangular coating accumulation model is selected, and the three-dimensional coating thickness accumulation distribution model is derived. The model has high accuracy, but also because of the limitation of its application requirements, the model lacks versatility and generalization ability. In [20], the coating accumulation model was obtained by fitting the coating thickness data of the fixed-distance vertical spraying sample. The model establishment method is closer to the actual coating thickness distribution, but the data acquisition method makes the data have a large amount of experimental and insufficient data information, which affects the accuracy of the model.

Although abundant theoretical and practical experience has been obtained in the establishment of the coating accumulation model at home and abroad, the focus of the research is, on the one hand, on the process of establishing the model through experimental data. The data acquisition method is complicated and the data coverage is insufficient, which reduces the efficiency of data acquisition and affects the accuracy and generalization of the model. On the other hand, the existing coating accumulation models are mostly 2D models, and the information expression is relatively single, which is difficult to accurately reflect the depth information and spatial relationship with the actual spraying so that the model has insufficient generalization ability. In repair spraying, due to the complexity and low repeatability of the sample structure, the spraying process simulation becomes an important part before the repair. The coating accumulation model established by the above method has the problems of low precision and weak generalization, which cannot solve the accuracy and reliability of the current spraying process simulation, thus affecting the accuracy of the repair spraying.

Therefore, this paper proposes a method for establishing a 3D coating accumulation model based on oblique spraying. By establishing an oblique spraying layer accumulation model to collect coating thickness data at different spray distances, a 3D coating accumulation model with measurement-point center distance and spraying-distance information is established. This method can quickly collect high-density experimental data and improve modeling efficiency and model accuracy. The spatial depth information of the 3D coating cumulative model also greatly improves the generalization ability of the model, thus ensuring the performance of the repair coating and the efficiency of the repair process.

## 2. Establishing Model

### 2.1. Accumulation Model of Oblique Spray Coating

**Figure 1:**
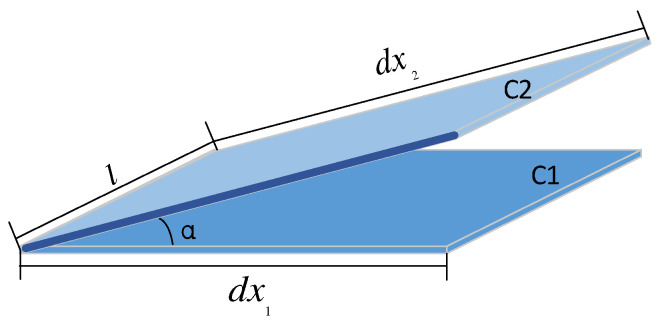
Cumulative model diagram of oblique spray coating.

It is assumed that there are two planes with the same spraying distance, as shown in Figure 1, where the *C*1 plane is perpendicular to the spray direction of the spray gun, and the angle α between the *C*2 plane and the plane *C*1 is the corresponding length infinitesimal, and the width is the same as l. If the flow rate is Q, the cumulative thickness of the coating is *G*1 and *G*2, respectively, then there is the following relationship:

(1) $$dx_{2}=\frac{dx_{1}}{cos\alpha }$$

The area S integral equation is

(2) $$S_{1}=\int_{a}^{b}ldx_{1}$$

(3) $$S_{1}=\int_{a}^{b}ldx_{2}$$

And in the ideal state, there exists $G=\frac{Q}{S}$*,* then there is

(4) $$G_{2}=G_{1}\times cos\alpha$$

There are many external factors affecting the coating thickness distribution, such as environmental temperature and humidity, paint viscosity, and pressure parameters, which affect the modeling accuracy of the coating accumulation model. The research focus of this paper is the modeling method. The external factors do not affect the validity and applicability of the modeling method proposed in this paper, and the method does not lose generality. Therefore, in the derivation of the cumulative model of the oblique spraying layer, the relationship between the spraying rate and the cumulative thickness of the coating is considered in this paper. Assuming the coating accumulation function G and the spraying rate v, the relationship between G and v is as follows:

(5) $$\frac{v_{1}}{v_{3}}=\frac{G_{3}}{G_{1}}$$

**Figure 2:**
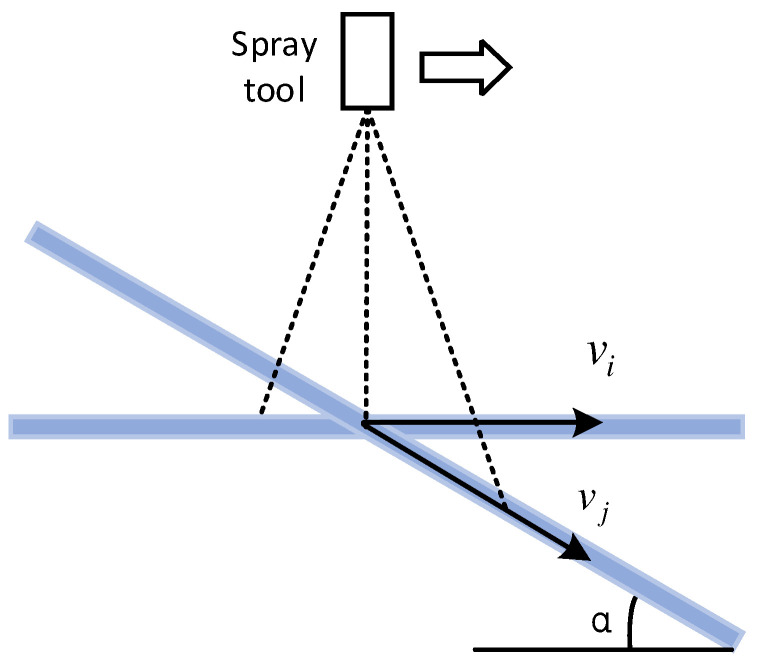
Paint drop point velocity analysis diagram (The dotted line is the spray amplitude schematic diagram).

Based on the above reasoning, when the spray gun is sprayed vertically, the spray gun moves at a rate, and the drop point speed of the paint is also $v_{i}$; when the trajectory direction of the spray gun is constant and the inclination angle of the sample is α, the falling point velocity of the paint is $v_{j}$, as shown in Figure 2. There is the following relationship:

(6) $$v_{j}=\frac{v_{i}}{cos\alpha }$$

Then, when the spraying trajectory parameters are constant, the relationship between the cumulative thickness $G_{j}$ of the inclined spraying coating and the cumulative thickness $G_{i}$ of the vertical spraying coating is

(7) $$G_{i}=\frac{G_{j}}{cos\alpha }$$

Therefore, combined with Equations (4) and (7), when the spray distance on the inclined plane is $h_{s}$, the relationship between the cumulative thickness of the coating corresponding to the spray distance $G_{j}$ and the cumulative thickness of the coating vertically sprayed at the same spray distance $G_{i}$ is shown as follows:

(8) $$G_{i}=\frac{G_{j}}{cos^{2}\alpha }$$

### 2.2. Generation of Model Training Data

**Figure 3:**
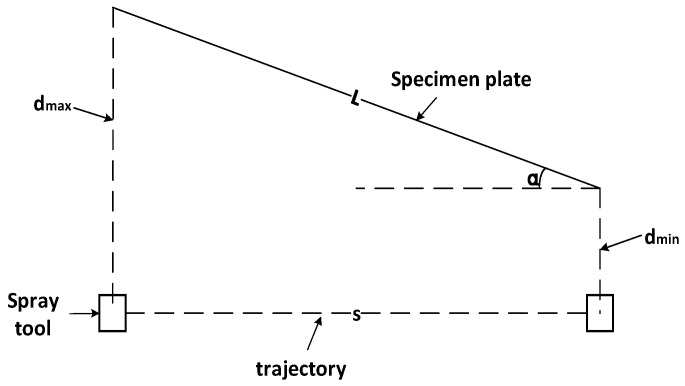
Oblique jet principle diagram.

The basic principle of the oblique spraying experiment is shown in Figure 3. The minimum spraying distance is $\;d_{min}$*,* the maximum spraying distance is $d_{max}$, and the length of the experimental sample is L. According to Equation (9), the rotation angle α of the specimen plate along the vertical axis is calculated. The spray gun is sprayed along the linear stroke. This experiment can obtain a gradually thickened linear coating, which is convenient for collecting the coating thickness distribution corresponding to different spraying distances.

(9) $$\alpha =arcsin\frac{d_{max}-d_{min}}{L}$$

**Figure 4:**
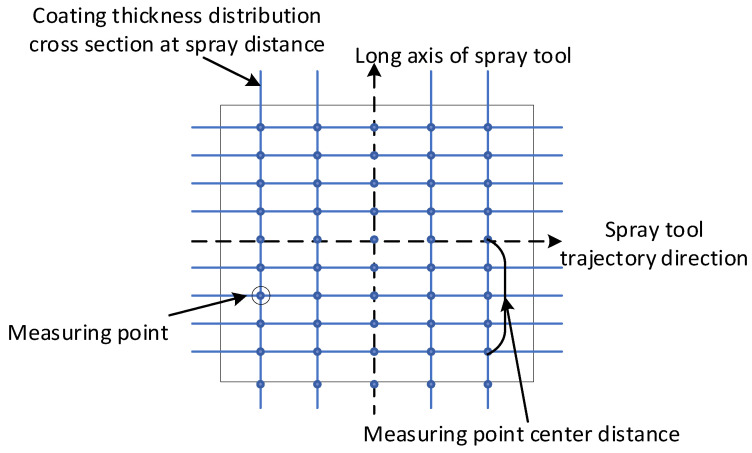
The distribution diagram of measurement points.

In order to obtain sufficient experimental data, this paper fully collects the data generated by the spraying experimental sample, as shown in Figure 4. Each data point contains two position information, the center distance of the measuring point and the spraying distance of the section where the measuring point is located.

### 2.3. 3D Coating Accumulation Model

The BP neural network is a multi-layer forward neural network based on an error backpropagation algorithm, which can realize any nonlinear mapping between input and output [21]. In this paper, the BP neural network with a double hidden layer structure is adopted. Each hidden layer can learn different levels of feature representation, so it can better deal with complex nonlinear problems, so as to improve the expression ability and fitting ability of the network.

The mathematical expression of the BP neural network model is as follows:

(10) $$Y=f_{a}(l^{T}\omega +b_{\theta })$$

where Y is the output neuron,  l  is the input neuron, ω  is the connection weight between neurons, $b_{\theta }\;$ is the threshold, and f is the neuron transfer function.

**Figure 5:**
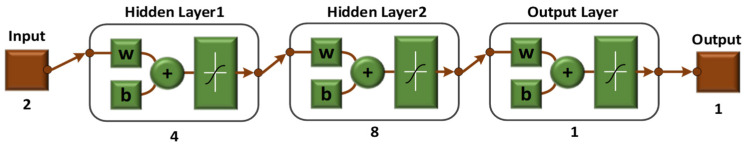
Neural network structure diagram.

In this paper, a BP neural network with a double hidden layer structure is used to predict the accumulation of coating. The structure is mainly composed of three parts: an input layer containing the center distance of the measuring point and the spraying distance of the section where the measuring point is located, an output layer containing the paint film thickness of the measuring point, and an intermediate hidden layer. The BP neural network structure is shown in Figure 5.

The transfer function, training function, and the number of hidden layer neurons used in the neural network structure need to be optimized to obtain the best performance model. Firstly, the transfer function includes the function from the input layer to the first hidden layer, the function between the hidden layers, and the function from the second hidden layer to the output layer. The optimal combination of transfer functions is selected by using the full-factor analysis method. Secondly, different training functions are selected to train the model and select the best. Finally, the number of double hidden layer neurons was determined by Equation (11), which was 4 and 8.

(11) $$N_{h}=\sqrt{N_{in}+N_{out}}+N_{a}$$

where $N_{h}$ is the number of hidden layer nodes, $N_{in}$ is the number of input layer nodes, $N_{out}$ is the number of output layer nodes, and $N_{a}$ is the adjustment constant between 1 and 10.

The performance of the neural network model is evaluated by R, as shown in Equation (12), which represents the relationship between the predicted value and the real value. Usually, the higher the R-value, the better the fitting effect of the model [22].

(12) $$R^{2}=1-\sum_{i=1}^{N}{\left(t_{i}-a_{i}\right)}^{2}/\sum_{i=1}^{N}{\left(t_{i}-\bar{t_{i}}\right)}^{2}$$

where $t_{i}$ is the actual value, $a_{i}$ is the predicted value, $\bar{t_{i}}$ is the average value of the actual value, and N is the number of data sets.

In the selection process of the transfer function and training function, the results of the use of each function are shown in Table 2 and Table 3.

It can be seen from Table 3 that when the transfer function is tansig-tansig-purelin, the R-value is the highest, so the model chooses this transfer function combination. In Table 3, the trainlm training function has the best performance, and the R-value is 0. 993. Therefore, the training function uses trainlm.

## 3. Verification Method

### 3.1. Verify the Oblique Spraying Coating Accumulation Model

In order to verify the accumulation model of the oblique spraying layer, an oblique spraying experiment and a fixed-distance spraying experiment are needed. The experimental samples of the two groups are L = 500 mm. The parameters of the spray gun were set as follows: spraying speed was 500 mm/s, atomization pressure was 0.2 MPa, fan control pressure was 0.1 MPa, and flow pressure was 0.15 MPa. The ambient temperature was 22.3 °C, the humidity was 41.2%, and the paint viscosity was 15.48 s.

The principle of the oblique spraying experiment is shown in Figure 3. The minimum spraying distance $d_{min}$ = 150 mm and the maximum spraying distance $d_{max}$ = 350 mm are set, and the thickness data of cross-section coatings with a spraying distance $h_{s}$ of 170 mm, 250 mm, and 290 mm are collected. In order to verify the reproducibility of the cumulative model of the oblique spraying layer, three oblique spraying experiments were performed under the same experimental conditions.

**Figure 6:**
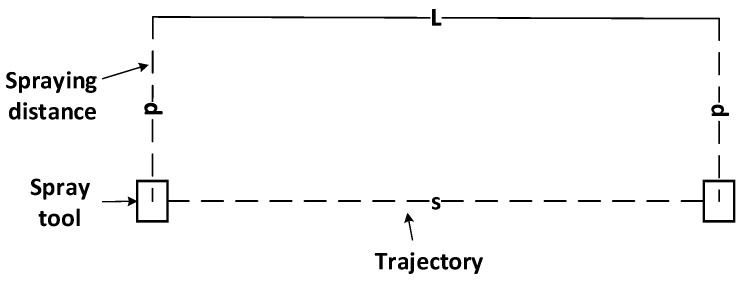
Spacing spraying sketch map.

The basic principle of the fixed-distance spraying experiment is shown in Figure 6. The axis of the spray gun is perpendicular to the plane specimen, and the spraying distance is kept constant along the straight-line stroke. This experiment can obtain a linear coating with the same thickness in the horizontal direction, which is convenient for analyzing the relationship between the distribution of the oblique spraying experimental coating and the fixed-distance experimental coating. The spraying distance $h_{s}$ is set to 170 mm, 250 mm, and 290 mm, and the corresponding coating thickness data are collected.

### 3.2. 3D Coating Accumulation Model Training and Verification

**Figure 7:**
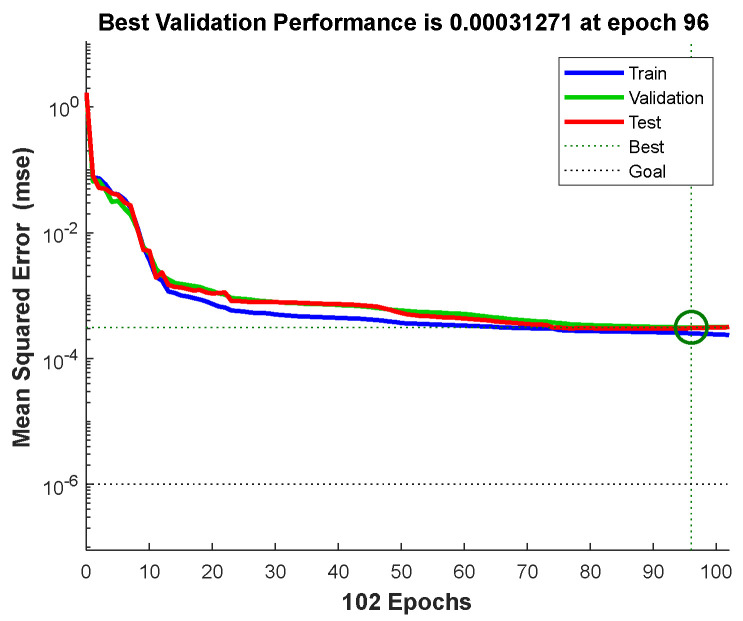
Network training situation (The green circle represents the number of iterations and the value of the network when the verification set has the best mean square error value).

**Figure 8:**
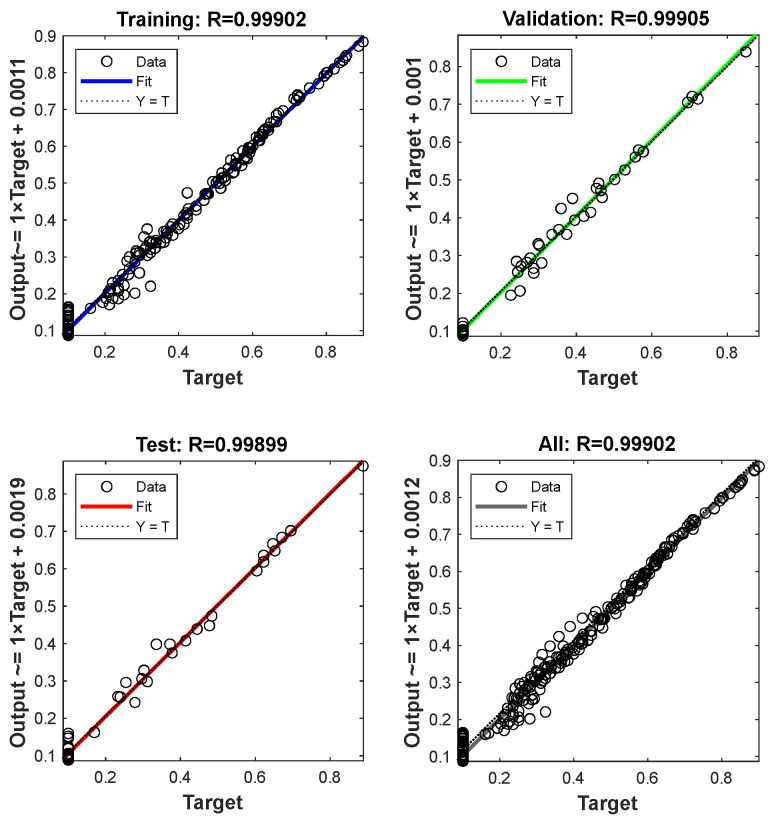
Network regression performance.

Taking the oblique spray test sample 1 as an example, through the data acquisition method of Figure 4, the coating thickness data with spraying distances of 170 mm, 190 mm, 230 mm, 250 mm, 270 mm, 290 mm, and 310 mm were collected on the oblique spray test sample 1 of Figure 6. The data on spraying distances of 170 mm, 250 mm, and 290 mm are shown in Table A1, Table A2 and Table A3 in Appendix A and the thickness data of other spraying distances are shown in Table 4. The above data are converted into fixed-distance spraying thickness data through the oblique spraying coating accumulation model Equation (9), which is used as the training sample data of the 3D coating accumulation model as shown in Figure 5. The data are randomly divided into a training set, verification set, and test set, accounting for 70%, 15%, and 15% of the total data set, respectively. The maximum number of iterations of the network is set to 2000, and the network converges after 96 times of learning, as shown in Figure 7. According to Equation (12), the R values of the training set, the validation set, and the test set are all greater than 0.99, and the model performance is good, as shown in Figure 8.

To verify the accuracy of the model after training the 3D coating accumulation model as shown in Figure 5, a spraying distance $h_{s}$ of 170 mm, 210 mm, and 250 mm were selected to carry out the single-track vertical spraying experiment, and 31 sets of data were measured. The obtained data were compared with the prediction results of the 3D coating accumulation model.

## 4. Results

### 4.1. Verification Results of Oblique Spray Coating Accumulation Model

**Figure 9:**
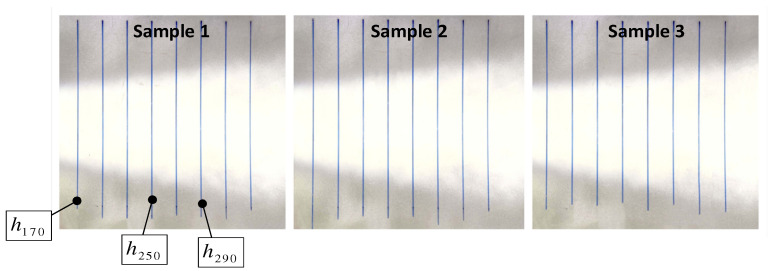
Oblique spray experimental samples, $d_{min}$ = 150 mm and $d_{max}$ = 350 mm. (The vertical line in the diagram is the mark of the measuring position).

**Figure 10:**
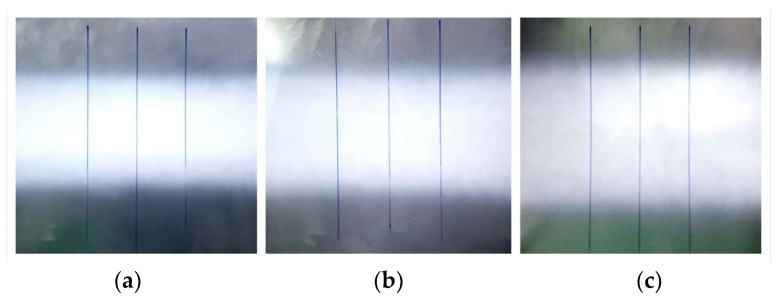
Sample diagram of fixed-distance spraying experiment. They should be listed as (**a**) spraying distance $h_{s}$ is 170 mm, (**b**) spraying distance $h_{s}$ is 250 mm, and (**c**) spraying distance $h_{s}$ is 290 mm. (The vertical line in the diagram is the mark of the measuring position).

**Figure 11:**
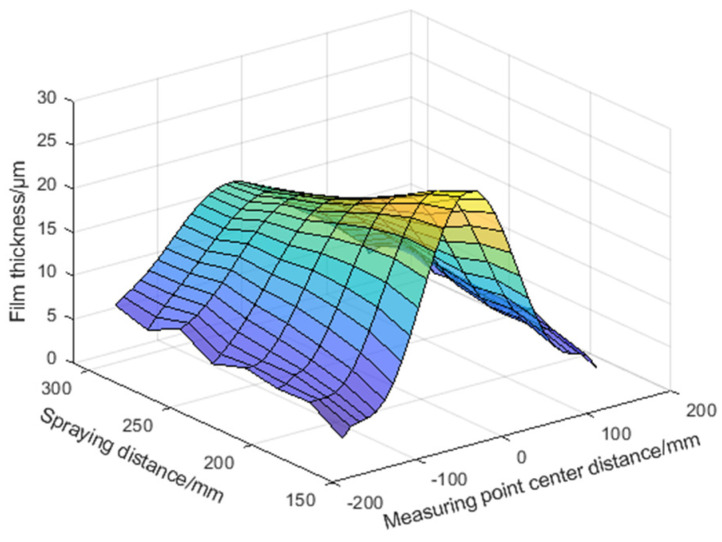
Coating thickness distribution of oblique spray test, $d_{min}$ = 150 mm$\;\mathrm{and}\;d_{max}$ = 350 m.

The three samples of the oblique spray experiment are shown in Figure 9. The prototype of the fixed-distance spraying experiment is shown in Figure 10. According to the data acquisition method of Figure 4, the coating thickness data are collected. The thickness distribution of the oblique spraying layer was drawn by taking the oblique spraying experiment sample 1 as an example, as shown in Figure 11. According to Equation (9), the rotation angle α of the specimen plate along the vertical axis can be calculated as follows:

$$\alpha =arcsin\frac{d_{max}-d_{min}}{L}=\frac{350\;mm-150\;mm}{500\;mm}=23.578^{{}^{\circ}}$$

**Figure 12:**
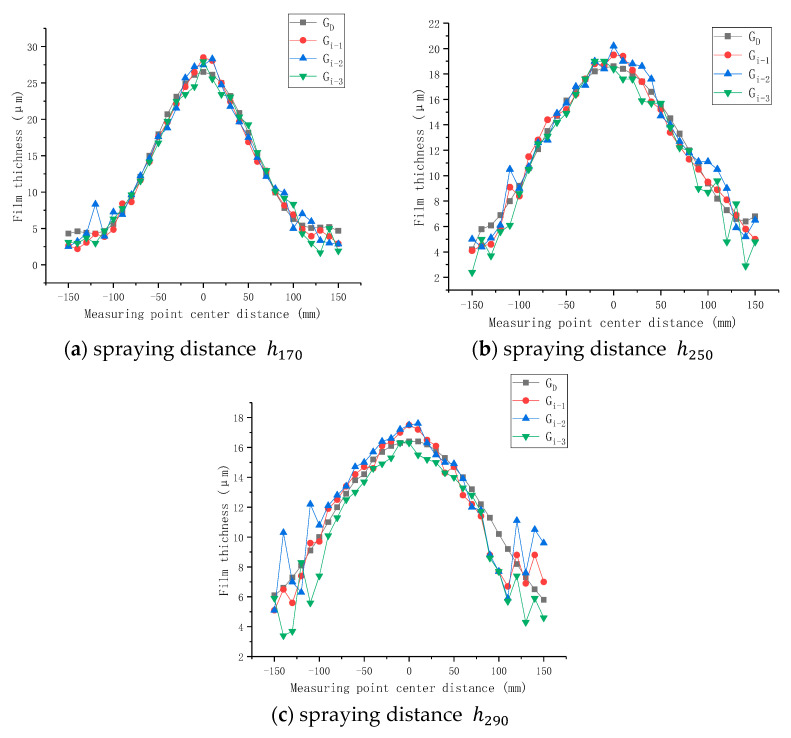
Comparison of coating thickness data between $G_{D}$ and $G_{i}$.

The coating thickness data of the three oblique spraying experiments are converted into $G_{i-1}$, $G_{i-2}$, and $G_{i-3}$ according to Equation (8), and the thickness of the fixed-distance spraying coating is $G_{D}$. The data are shown in Table A1, Table A2 and Table A3 in Appendix A. The calculated values of the model of each spray distance $G_{i-1}$, $G_{i-2}$, and $G_{i-3}$ and the change trend of the fixed-distance spraying $G_{D}$ are shown in Figure 12. The performance of the model is evaluated by the absolute error and relative error of the data.

### 4.2. Validation Results of 3D Coating Accumulation Model

**Figure 13:**
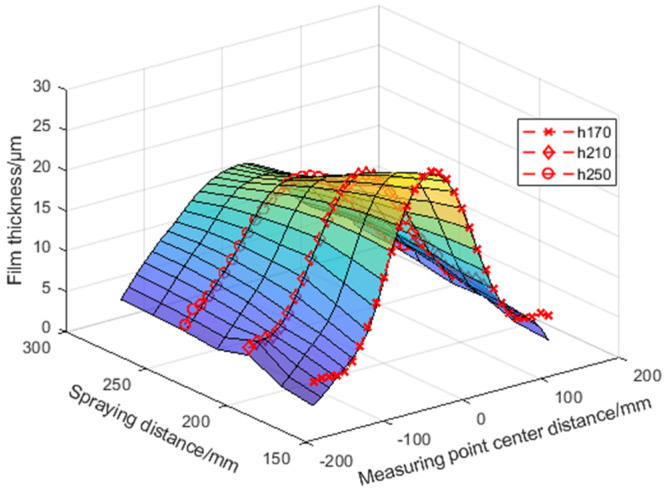
When the spraying distance is 170 mm, 210 mm, and 250 mm, the model prediction data are compared with the real data.

The experimental data are shown in Table A4, Table A5 and Table A6 in Appendix A. The thickness variation trend of the output value of the model and the actual value is shown in Figure 13.

## 5. Discussion

(1) For the performance of the oblique spraying layer accumulation model, it can be seen from the coating surface quality of the oblique spraying experimental template in Figure 9 and the fixed-distance spraying experimental template in Figure 10, there is no sagging phenomenon on the surface of the sample with the minimum spraying distance of 170 mm, and the coating coverage is completed on the surface of the sample with the maximum spraying distance of 290 mm, which conforms to the distribution law of the middle thick edge thin. It can be seen from Figure 12 that the calculated value of the model is consistent with the thickness change trend of the actual value. It can be seen from Table A1, Table A2 and Table A3 in Appendix A that the absolute errors of the three spray distances are −1.3~2 μm, −1.9~2.2 μm, and −1.6~1.4 μm, respectively, when the center distance of the measuring point is in the range of −80~80 mm. The absolute errors and the relative errors of the three spray distances are large when the center distance of the measuring point is in the range of −150~−90 mm and 90~150 mm. This is because the coating in the edge area of the spray is thin, and a small thickness difference will also produce a large relative error. From Table 5, the relative error is small when the center distance of the measurement point is in the range of 80~80 mm, and the average relative error of the same spray distance is relatively close on the three oblique spray test samples. The range of average relative error is (4.22 ± 0.21)% when the spray distance is 170 mm, the range of average relative error is (3.78 ± 0.08)% when the spray distance is 250 mm, and the range of average relative error is (4.29 ± 0.07)% when the spray distance is 290 mm.
(2) The performance of the 3D coating accumulation model can be seen in Table A4, Table A5 and Table A6 in Appendix A. Within the center distance of the measurement point of −150~150 mm, the absolute errors of the coating thickness predicted by the model for the spraying distance of 170 mm, 210 mm, and 250 mm are −0.9~2.4 μm, −0.5~1.8 μm, and 0~1.2 μm, respectively, and the average relative errors are 9.7%, 3.7%, and 4.5%, respectively. The center distance of the measuring point is close to the edge of the spray, such as the center distance of the measuring point is −150 mm and 150 mm, the error and relative error become larger, because in the edge part, the coating quality decreases and the thickness is not uniform, which affects the prediction accuracy of the model. According to Figure 13, the expression of the thickness change trend of the output value of the model is consistent with the actual value in space.

The results of this paper are compared with similar references, as shown in Table 6. Compared with Reference [6], the average absolute error of this paper is increased by 0.61 μm, and the average relative error is increased by 1.2%. Compared with Reference [13], the average relative error of this paper is increased by 21.5%.

## 6. Conclusions

In this paper, a method for establishing a 3D coating accumulation model based on oblique spraying is proposed. After experimental verification, the following conclusions are drawn:

(1) A method for establishing the accumulation model of oblique spraying coating was proposed. This method can realize the rapid acquisition of coating thickness data at any spray distance within the range of spray distance set by the experimental sample and has efficient data acquisition ability and the ability to extract high-density valuable information.
(2) In the range of spraying distance 170~290 mm, the average relative error of the model is less than 4.1%, which can realize the efficient generation of training data of the 3D coating cumulative model. The model has good applicability and accuracy in the range of 80~80 mm from the center distance of the measurement point.
(3) In this paper, a 3D coating accumulation model with coating as a function is established with spraying distance and spraying position as variables. The accuracy of the model is 1.2% and 21.5% higher than that of the two similar models. Using this model, the coating thickness can be accurately calculated according to the spraying distance and spraying position, so as to improve the coating simulation accuracy in the spraying simulation software.

## Figures and Tables

**Table 2:** Combined regression performance of transfer function.

Transfer Function	R	Transfer Function	R	Transfer Function	R
1-1-1	0.914	2-1-1	0.97	3-1-1	0.967
1-1-2	0.073	2-1-2	0.729	3-1-2	0.641
1-1-3	0.993	2-1-3	0.859	3-1-3	0.891
1-2-1	0.955	2-2-1	0.912	3-2-1	0.956

Note. 1-tansig, 2-logsig, 3-purelin.

**Table 3:** Training function regression performance.

Training Function	R	Training Function	R
Trainlm	0.993	Traincgf	0.86
Trainbr	0.987	Traincgp	0.987
Trainbfg	0.261	Trainoss	0.973
Trainrp	0.98	Traingdx	0.871
Trainscg	0.986	Traingdm	0.982
Traincgb	0.929	traingd	0.983

**Table 4:** Three-dimensional coating cumulative model training part of the data.

Measuring Point Center Distance/mm	h_190_/μm	h_230_/μm	h_270_/μm	h_310_/μm
−80	10.8	12.2	12.5	11.3
−70	13.3	14.1	13.7	12.4
−60	15.8	15.8	14.8	13.4
−50	18.3	17.3	15.8	14.3
−40	20.6	18.7	16.6	15.0
−30	22.5	19.7	17.2	15.5
−20	23.9	20.3	17.5	15.8
−10	24.7	20.6	17.6	15.8
0	24.8	20.6	17.5	15.5
10	24.4	20.1	17.2	15.1
20	23.3	19.3	16.7	14.4
30	21.7	18.2	16.0	13.7
40	19.7	16.9	15.1	12.8
50	17.4	15.4	14.2	11.9
60	15.1	13.8	13.3	11.1
70	12.7	12.1	12.4	10.3
80	10.6	10.6	11.5	9.7

**Table 5:** The average relative error of the center distance of the measuring point is −80~80 mm.

Spraying Distance	*G_i_*_−1_	*G_i_*_−2_	*G_i_*_−3_	Average
h_170_	4.43%	4.23%	4.01%	4.22%
h_250_	3.85%	3.80%	3.70%	3.78%
h_290_	4.22%	4.31%	4.35%	4.29%
Average	--	--	--	4.09%

**Table 6:** The average absolute error and average relative error between the simulation results and the experimental results in the same literature.

Author and Year of Publication	Average Absolute Error	Average Relative Error
This work	0.31 μm	3.7%
Reference [6]: Guo lei, W. 2017	0.92 μm	4.9%
Reference [13]: Chen, C. 2016	--	25.2%

## Data Availability

The data presented in this study are available on request from the corresponding author.

## Appendix A

**Table A1:** Comparison of coating thickness data of spraying distance *h*_170_.

Measuring Point Center Distance/mm	$G_{D}$/μm	$G_{i-1}$/μm	$G_{i-2}$/μm	$G_{i-3}$/μm	Absolute Error/μm			Relative Error/%		
−150	4.3	2.6	2.5	3.1	−1.7	−1.8	−1.2	38.89	41.66	27.77
−140	4.6	2.2	3.2	3.0	−2.4	−1.4	−1.6	52.47	30.00	35.19
−130	4.4	3.1	4.3	3.7	−1.4	−0.1	−0.7	30.70	2.80	16.30
−120	4.3	4.2	8.3	3.0	−0.1	4.0	−1.3	1.51	93.29	30.97
−110	4.6	3.8	3.9	4.6	−0.8	−0.7	0.0	17.31	15.60	0.25
−100	5.6	4.8	7.3	6.3	−0.8	1.6	0.7	13.82	29.27	12.32
−90	7.2	8.4	6.9	7.7	1.2	−0.3	0.5	16.24	4.60	6.92
−80	9.4	8.6	9.6	9.6	−0.8	0.2	0.2	8.42	2.08	2.08
−70	12.1	11.6	12.2	11.5	−0.5	0.2	−0.6	3.91	1.33	4.57
−60	15.0	14.2	14.6	14.2	−0.8	−0.4	−0.8	5.61	2.44	5.61
−50	17.9	17.6	17.6	16.8	−0.3	−0.3	−1.2	1.88	1.88	6.52
−40	20.7	19.6	18.8	19.7	−1.1	−1.9	−1.0	5.43	9.26	4.66
−30	23.1	22.1	21.5	22.5	−1.0	−1.6	−0.6	4.23	6.81	2.69
−20	24.9	24.5	25.7	23.4	−0.5	0.7	−1.5	1.93	3.00	6.06
−10	26.1	26.5	27.2	24.5	0.4	1.1	−1.6	1.44	4.33	6.15
0	26.5	28.5	27.5	27.9	2.0	1.0	1.4	7.51	3.62	5.41
10	26.1	28.1	28.3	25.6	1.9	2.2	−0.6	7.39	8.30	2.17
20	25.0	25.0	24.7	23.4	0.0	−0.3	−1.6	0.13	1.08	6.31
30	23.2	22.4	21.8	23.1	−0.8	−1.4	−0.1	3.32	6.22	0.58
40	20.9	19.7	19.6	20.3	−1.2	−1.2	−0.5	5.60	5.97	2.56
50	18.2	16.9	17.5	19.3	−1.3	−0.7	1.1	7.04	3.77	6.05
60	15.3	14.2	14.7	15.5	−1.1	−0.6	0.2	7.24	3.61	1.06
70	12.5	12.9	12.1	13.0	0.4	−0.4	0.5	3.54	2.81	3.86
80	9.9	10.0	10.5	10.1	0.1	0.5	0.2	0.66	5.45	1.86
90	7.8	8.2	9.9	9.2	0.4	2.1	1.3	4.52	26.34	17.20
100	6.3	6.9	5.0	8.3	0.6	−1.3	2.0	9.81	20.48	32.54
110	5.4	4.9	7.0	4.3	−0.5	1.6	−1.1	8.71	30.31	20.49
120	5.1	3.9	5.9	3.0	−1.1	0.9	−2.1	22.58	17.31	41.35
130	5.1	4.7	3.3	1.7	−0.4	−1.8	−3.5	8.16	35.17	67.59
140	5.2	3.9	3.0	5.0	−1.3	−2.2	−0.2	25.39	42.90	4.07
150	4.7	2.9	2.9	1.9	−1.8	−1.8	−2.8	38.12	38.97	59.31

**Table A2:** Comparison of coating thickness data of spraying distance *h*_250_.

Measuring Point Center Distance/mm	$G_{D}$/μm	$G_{i-1}$/μm	$G_{i-2}$/μm	$G_{i-3}$/μm	Absolute Error/μm			Relative Error/%		
−150	4.2	4.1	5.0	2.4	0.0	0.8	−1.8	0.66	20.35	42.69
−140	5.8	4.5	4.4	5.0	−1.3	−1.4	−0.8	22.41	24.45	14.24
−130	6.1	4.6	5.1	3.7	−1.5	−1.0	−2.4	24.22	16.45	39.77
−120	6.9	5.9	6.1	5.6	−1.0	−0.8	−1.3	14.13	11.82	18.74
−110	8.0	9.1	10.5	6.1	1.2	2.5	−1.9	14.55	31.48	23.80
−100	9.2	8.4	9.0	8.6	−0.8	−0.2	−0.7	9.13	2.27	7.42
−90	10.6	11.5	10.7	10.5	0.8	0.1	−0.2	7.98	0.54	1.70
−80	12.1	12.8	12.6	12.5	0.7	0.5	0.4	5.75	4.44	3.45
−70	13.5	14.4	12.8	13.1	1.0	−0.6	−0.4	7.26	4.52	2.76
−60	14.7	14.8	14.9	14.2	0.1	0.1	−0.6	0.60	0.87	3.97
−50	15.9	15.2	15.7	14.9	−0.7	−0.2	−1.0	4.15	1.16	6.40
−40	16.8	16.4	17.0	16.4	−0.4	0.2	−0.4	2.60	0.93	2.60
−30	17.6	17.6	17.1	17.6	−0.1	−0.5	0.0	0.31	2.79	0.09
−20	18.2	18.8	19.0	19.0	0.6	0.9	0.9	3.46	4.77	4.77
−10	18.5	18.7	18.4	19.0	0.2	0.0	0.6	1.05	0.24	2.98
0	18.6	19.5	20.2	18.4	0.9	1.7	−0.1	5.10	8.95	0.66
10	18.4	19.4	19.0	17.6	1.0	0.6	−0.8	5.35	3.41	4.34
20	18.0	18.3	18.8	17.6	0.3	0.8	−0.4	1.66	4.30	2.30
30	17.4	17.4	18.6	15.9	0.0	1.1	−1.5	0.17	6.54	8.48
40	16.6	15.8	17.6	15.7	−0.8	1.0	−0.9	5.02	5.96	5.50
50	15.6	15.2	14.7	15.7	−0.4	−0.9	0.1	2.63	5.67	0.41
60	14.5	13.4	14.0	13.8	−1.1	−0.5	−0.7	7.37	3.27	4.91
70	13.3	12.3	12.7	12.2	−1.0	−0.5	−1.0	7.41	4.12	7.71
80	12.0	11.3	11.8	11.97	−0.7	−0.3	−0.2	5.61	2.63	1.64
90	10.7	10.5	11.1	9.0	−0.2	0.4	−1.6	1.75	3.83	15.15
100	9.4	9.5	11.1	8.7	0.1	1.7	−0.7	1.41	17.89	7.46
110	8.2	8.9	10.5	9.6	0.6	2.2	1.4	7.88	27.14	17.03
120	7.3	8.1	9.0	4.8	0.8	1.8	−2.5	11.53	24.04	34.72
130	6.6	6.9	5.9	7.8	0.3	−0.7	1.2	4.37	10.54	18.09
140	6.4	5.8	5.2	2.9	−0.6	−1.2	−3.6	9.95	18.59	55.59
150	6.8	5.0	6.5	4.8	−1.7	−0.2	−2.0	25.54	3.26	29.65

**Table A3:** Comparison of coating thickness data of spraying distance *h*_290_.

Measuring Point Center Distance/mm	$G_{D}$/μm	$G_{i-1}$/μm	$G_{i-2}$/μm	$G_{i-3}$/μm	Absolute Error/μm			Relative Error/%		
−150	6.1	5.1	5.1	5.9	−1.0	−1.0	−0.2	17.01	16.36	2.75
−140	6.6	6.5	10.3	3.4	−0.1	3.8	−3.1	1.34	57.02	47.66
−130	7.3	5.6	7.0	3.7	−1.7	−0.3	−3.6	23.20	3.59	49.35
−120	8.1	7.4	6.3	8.3	−0.7	−1.8	0.2	8.69	22.37	2.54
−110	9.1	9.6	12.2	5.6	0.5	3.2	−3.5	5.48	35.24	38.29
−100	10.0	9.7	10.8	7.4	−0.3	0.8	−2.7	3.33	7.72	26.61
−90	11.0	11.9	12.1	10.1	0.8	1.1	−0.9	7.65	9.81	8.50
−80	12.0	12.5	12.8	11.3	0.5	0.8	−0.7	3.85	6.82	6.04
−70	12.9	13.4	13.4	12.5	0.5	0.5	−0.5	3.78	3.78	3.57
−60	13.8	14.2	14.7	13.0	0.4	0.9	−0.8	2.57	6.88	6.05
−50	14.2	14.7	15.0	13.7	0.5	0.8	−0.5	3.70	5.37	3.83
−40	15.2	14.6	15.7	14.6	−0.6	0.5	−0.6	3.96	3.35	3.70
−30	15.7	16.1	16.4	14.9	0.4	0.7	−0.8	2.49	4.51	5.33
−20	16.1	16.4	16.6	15.3	0.3	0.6	−0.7	1.80	3.52	4.61
−10	16.3	17.0	17.2	16.3	0.7	0.9	0.0	4.20	5.66	0.17
0	16.4	17.5	17.5	16.3	1.1	1.1	−0.1	6.52	6.52	0.73
10	16.4	17.2	17.6	15.5	0.9	1.2	−0.9	5.43	7.61	5.48
20	16.2	16.5	16.3	15.2	0.4	0.1	−0.9	2.33	0.86	5.77
30	15.8	16.1	15.5	15.0	0.3	−0.3	−0.8	2.06	2.20	5.21
40	15.3	14.3	15.0	14.3	−1.1	−0.3	−1.1	6.88	2.23	6.88
50	14.7	14.7	14.9	14.0	0.0	0.2	−0.7	0.22	1.03	4.63
60	14.0	12.8	13.9	13.3	−1.2	−0.1	−0.7	8.45	0.51	4.76
70	13.2	12.2	12.0	12.8	−0.9	−1.1	−0.3	6.88	8.69	2.36
80	12.2	11.4	11.8	11.7	−0.8	−0.5	−0.6	6.70	3.79	4.76
90	11.3	8.8	8.8	8.6	−2.4	−2.5	−2.7	21.47	21.82	23.93
100	10.2	7.7	7.7	7.7	−2.5	−2.5	−2.5	24.88	24.49	24.49
110	9.2	6.7	5.9	5.7	−2.5	−3.3	−3.5	26.84	35.45	38.03
120	8.2	8.8	11.1	7.4	0.5	2.8	−0.8	6.67	34.67	10.22
130	7.3	6.9	7.6	4.3	−0.4	0.3	−3.0	5.84	4.50	41.22
140	6.5	8.8	10.5	5.9	2.3	4.0	−0.5	35.99	61.72	8.12
150	5.8	7.0	9.6	4.6	1.2	3.8	−1.2	20.33	65.19	20.46

**Table A4:** Experimental data on spraying distance *h*_170_.

Measuring Point Center Distance/mm	Coating Thickness/μm		Absolute Error/μm	Relative Error/%
	True Value	Predict Value		
−150	4.3	2	2.3	53.3
−140	4.6	3.1	1.5	32.4
−130	4.4	3.4	1.0	22.8
−120	4.3	3.6	0.7	16.4
−110	4.6	4	0.6	14.0
−100	5.6	5.1	0.5	9.1
−90	7.2	6.7	0.5	7.3
−80	9.4	9	0.4	4.6
−70	12.1	11.7	0.4	3.2
−60	15.0	14.6	0.4	2.6
−50	17.9	17.7	0.2	1.3
−40	20.7	20.5	0.2	1.0
−30	23.1	23.6	−0.5	2.2
−20	24.9	25.3	−0.4	1.4
−10	26.1	25.9	0.2	0.8
0	26.5	26.1	0.4	1.6
10	26.1	25.5	0.6	2.4
20	25.0	25.1	−0.1	0.4
30	23.2	23.8	−0.6	2.6
40	20.9	21.8	−0.9	4.5
50	18.2	18.5	−0.3	1.8
60	15.3	15.1	0.2	1.3
70	12.5	12.3	0.2	1.4
80	9.9	9.8	0.1	1.3
90	7.8	7.6	0.2	2.7
100	6.3	6.1	0.2	2.9
110	5.4	5.1	0.3	5.3
120	5.1	4.7	0.4	7.3
130	5.1	4.4	0.7	14.3
140	5.2	3.8	1.4	27.0
150	4.7	2.3	2.4	50.8
Average				9.7

**Table A5:** Experimental data on spraying distance $h_{210}$.

Measuring Point Center Distance/mm	Coating Thickness/μm		Absolute Error/μm	Relative Error
	True Value	Predict Value		
−150	4.9	4.0	0.89	18.1
−140	5.3	5.5	0.19	3.6
−130	5.6	5.5	0.10	1.8
−120	5.6	5.8	0.10	1.8
−110	6.5	6.6	0.11	1.7
−100	7.9	8.0	0.13	1.6
−90	9.7	9.8	0.13	1.3
−80	11.7	11.8	0.11	1.0
−70	13.9	14.0	0.10	0.7
−60	16.0	16.1	0.07	0.4
−50	18.1	18.1	0.03	0.2
−40	19.8	19.8	0.02	0.1
−30	21.2	21.2	0.06	0.3
−20	22.2	22.1	0.06	0.3
−10	22.7	22.7	0.00	0.0
0	22.6	22.7	0.14	0.6
10	22.0	22.4	0.31	1.4
20	21.0	21.5	0.48	2.3
30	19.6	20.2	0.58	3.0
40	17.9	18.5	0.63	3.5
50	15.9	16.5	0.64	4.0
60	13.9	14.5	0.67	4.8
70	12.5	12.6	0.08	0.6
80	11.2	10.8	0.37	3.3
90	10.0	9.3	0.70	7.0
100	8.4	8.0	0.37	4.4
110	7.5	7.0	0.55	7.3
120	6.9	6.3	0.69	9.9
130	6.1	5.9	0.18	3.0
140	5.7	5.7	0.00	0.1
150	4.6	3.4	1.20	26.1
Average				3.7

**Table A6:** Experimental data on spraying distance $h_{250}$.

Measuring Point Center Distance/mm	Coating Thickness/μm		Absolute Error/μm	Relative Error
	True Value	Predict Value		
−150	4.15	3.7	0.5	10.8
−140	5.82	4.6	1.2	21.0
−130	6.12	5.5	0.6	10.1
−120	6.88	6.6	0.3	4.0
−110	7.96	7.8	0.2	2.0
−100	9.25	9.2	0.0	0.5
−90	10.65	10.7	−0.1	0.5
−80	12.07	12.2	−0.1	1.1
−70	13.45	13.6	−0.1	1.1
−60	14.74	15	−0.3	1.8
−50	15.88	16.3	−0.4	2.6
−40	16.85	17.3	−0.5	2.7
−30	17.62	18.1	−0.5	2.8
−20	18.16	18.7	−0.5	3.0
−10	18.48	18.9	−0.4	2.3
0	18.56	18.9	−0.3	1.9
10	18.40	18.5	−0.1	0.5
20	18.01	18	0.0	0.1
30	17.41	17.1	0.3	1.8
40	16.61	16.1	0.5	3.1
50	15.63	15	0.6	4.0
60	14.51	13.8	0.7	4.9
70	13.27	12.6	0.7	5.1
80	11.97	11.5	0.5	3.9
90	10.65	10.4	0.3	2.4
100	9.38	9.4	0.0	0.2
110	8.23	8.6	−0.4	4.5
120	7.29	7.8	−0.5	7.0
130	6.65	7	−0.4	5.3
140	6.43	6.2	0.2	3.5
150	6.76	5	1.8	26.1
Average				4.5

## References

[1] Antonio J.K., Ramabhadran R., Ling T.L. (1997). A framework for optimal trajectory planning for automated spray coating. Int. J. Robot. Autom..

[2] Tokunaga H., Okano T., Matsuki N., Tanaka F., Kishinami T. A Method to Solve Inverse Kinematics Problems Using Lie Algebra and Its Application to Robot Spray Painting Simulation. Proceedings of the ASME 2004 International Design Engineering Technical Conferences and Computers and Information in Engineering Conference.

[3] Zeng Y., Zhang Y., He J., Zhou H., Zhang C., Zheng L. (2016). Prediction model of coating growth rate for varied dip-angle spraying based on gaussian sum model. Math. Probl. Eng..

[4] Zhang M. (2006). Brief Introduction of Film Thickness Control During Paint Spraying by Robot. Xiandai Tuliao Yu Tuzhuang.

[5] Arkan M.A.S., Balkan T. (2000). Process modeling, simulation, and paint thickness measurement for robotic spray painting. J. Robot. Syst..

[6] Yonggui Z., Yumei H., Feng G., Wei W. (2006). New model for air spray gun of robotic spray-painting. J. Mech. Eng..

[7] Suh S.-H., Woo I.-K., Noh S.-K. Development of an automatic trajectory planning system (ATPS) for spray painting robots. Proceedings of the 1991 IEEE International Conference on Robotics and Automation.

[8] Liu X.-M., Liu T., Yang L.-S. (2018). Spray painting film thickness distribution on panel and optimization of width of paint film overlay. Surf. Technol..

[9] Wang G., Yi Q., Miao D., Chen K., Wang L. (2017). Multivariable coating thickness distribution model for robotic spray painting. J. Tsinghua Univ. (Sci. Technol.).

[10] Zhou Y., Ma S., Li A., Yang L. (2019). Path planning for spray painting robot of horns surfaces in ship manufacturing. IOP Conf. Ser. Mater. Sci. Eng..

[11] Nieto Bastida S., Lin C.-Y. (2023). Autonomous Trajectory Planning for Spray Painting on Complex Surfaces Based on a Point Cloud Model. Sensors.

[12] Yi W., Guolei W., Bo L., Jiyu Y., Dunmin L. (2022). Analysis of Coating Uniformity in Boundary Zone of Surface Spraying with Large-size. Mech. Sci. Technol. Aerosp. Eng..

[13] Qi S., Zheng Q., Zhao C., Sun Y., Xia H., Jiang D. (2023). Research on Trajectory Planning of Spraying Robot Based on Coating Thickness Model. Mach. Tool Hydraul..

[14] Shi T., Xu J., Cui J., Tao L., Xu W., Wang Z., Ji J. (2023). Variable Velocity Coating Thickness Distribution Model for Super-Large Planar Robot Spraying. Coatings.

[15] Hua X., Zhang S., Liu X., Chen Z., Wang G., Chen K. (2020). Optimization of spraying trajectory based on elliptical double β spraying gun model. J. Tsinghua Univ. (Sci. Technol.).

[16] Zeng Y., Yu Y., Zhao X., Liu Y., Liu J., Liu D. (2021). Trajectory Planning of Spray Gun With Variable Posture for Irregular Plane Based on Boundary Constraint. IEEE Access.

[17] Chen C., Xie Y., Verdy C., Liao H., Deng S. (2016). Modelling of coating thickness distribution and its application in offline programming software. Surf. Coat. Technol..

[18] Wu H., Xie X., Liu M., Chen C., Liao H., Zhang Y., Deng S. (2019). A new approach to simulate coating thickness in cold spray. Surf. Coat. Technol..

[19] Hua R.-X., Zou W., Chen G.-D., Ma H.-X., Zhang W. (2021). A model of spray tool and a parameter optimization method for spraying path planning. Int. J. Autom. Comput..

[20] Gleeson D., Jakobsson S., Salman R., Sandgren N., Edelvik F., Carlson J.S., Lennartson B. Robot spray painting trajectory optimization. Proceedings of the 2020 IEEE 16th International Conference on Automation Science and Engineering (CASE).

[21] Zhang P., Liu J., Chen C., Li Y.Q. The algorithm study for using the back propagation neural network in CT image segmentation. Proceedings of the International Conference on Innovative Optical Health Science.

[22] Yu D., Su C., Yang D., Bao H. Prediction of spraying process parameters based on BP neural network. Proceedings of the 2022 12th International Conference on CYBER Technology in Automation, Control, and Intelligent Systems (CYBER).

